# The Price of Exposure: Xeroderma Pigmentosum and Skin Cancer

**DOI:** 10.7759/cureus.85703

**Published:** 2025-06-10

**Authors:** Savi Aneja, Mala Bhalla, Tanya Jain

**Affiliations:** 1 Dermatology, Venerology and Leprosy, Government Medical College & Hospital, Chandigarh, Chandigarh, IND; 2 Dermatology, Government Medical College & Hospital, Chandigarh, Chandigarh, IND

**Keywords:** autosomal recessive disease, genetic syndromes, squamous cell carcinoma (scc), sun exposure, xeroderma pigmentosum

## Abstract

Xeroderma pigmentosum (XP) is a rare autosomal recessive genodermatosis characterized by a defect in the nucleotide excision repair pathway of DNA. This condition leads to extreme photosensitivity, pigmentary changes, premature aging, and increased risk of UV-induced skin and mucous membrane neoplasms. We report a case of squamous cell carcinoma in a 55-year-old female with XP. A 55-year-old female presented with a single asymptomatic skin lesion on the left cheek that had developed over the past two years. Examination revealed a well-defined, round to oval black raised nodulo-plaque, with a central adherent crust and raised superior margin. Histopathological examination revealed an invasive tumor arising from hyperplastic and dysplastic squamous epithelium with cytokeratin positivity. XP presents significant clinical challenges due to its severe photosensitivity and the associated risk of developing multiple skin cancers at a young age, which highlights the necessity for ongoing research into effective treatments and preventive strategies. Genetic counseling can play a vital role for families, enabling them to understand the implications for future generations. Increased awareness can help improve the quality of life and reduce the incidence of associated malignancies.

## Introduction

Xeroderma pigmentosum (XP) is a rare, inherited skin disorder caused by a defect in the DNA repair system, specifically the nucleotide excision repair pathway, which normally repairs damage from ultraviolet (UV) light [1]. This impairment leads to extreme sensitivity to sunlight, changes in skin pigmentation, early-onset skin aging, eye damage, and a dramatically increased risk of UV-induced skin and mucous membrane cancers. In some cases, patients may also develop progressive neurological deterioration. Globally, XP occurs in approximately one in one million births, with higher prevalence in regions with consanguineous marriages, such as parts of North Africa, the Middle East, and Japan.

Individuals with XP face a 10,000-fold higher risk of non-melanoma skin cancers (NMSC) and a 2,000-fold increase in melanoma risk [2]. Among NMSCs, squamous cell carcinoma (SCC) is especially concerning, commonly developing in sun-exposed areas. A large case series and literature review by Baykal et al. found SCC in 25% of XP patients, underscoring its clinical significance [3]. Life expectancy varies significantly depending on neurological involvement: patients with neurodegeneration have a median lifespan of about 29 years, while those without may live up to 37 years [4].

Despite these risks, some individuals with XP survive well beyond typical expectations. We report a case of SCC in a 55-year-old woman with XP, an unusual presentation both because of her extended survival and as a reminder that the risk of UV-related cancers persists lifelong, even in rare long-term survivors. 

## Case presentation

**Figure 1:**
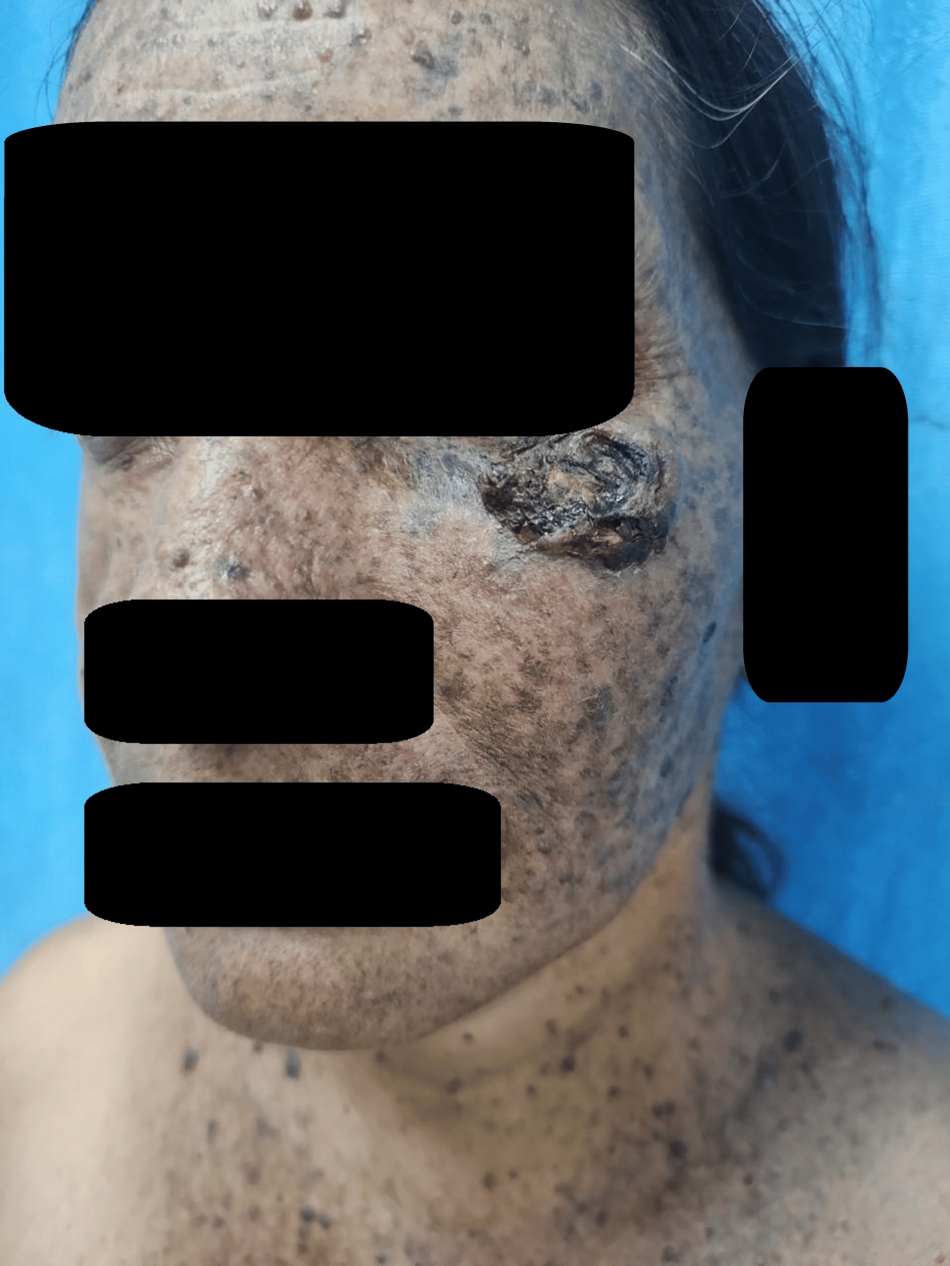
A well-defined, 4 x 4 cm black nodulo-plaque with a round to oval shape, featuring a central adherent crust on the left cheek

A 55-year-old female from Una, Himachal Pradesh, presented with a single asymptomatic skin lesion on her left cheek that had developed over the past two years. Examination revealed a well-defined, round to oval black raised nodulo-plaque approximately 4 x 4 cm in diameter, with a central adherent crust and skin-colored to erythematous raised superior margin (Figure 1).

The lesion was firm to hard, non-tender, non-mobile, and adherent to underlying structures. Regional lymph nodes were not enlarged.

Additional findings included multiple hyperpigmented macules and papules on sun-exposed areas, primarily the face, neck, upper back, and dorsum of hands and feet. The patient noticed these lesions over time, which gradually appeared on areas frequently exposed to sunlight. These hyperpigmented spots, common in XP, are often one of the earliest signs of the condition and can progressively worsen with continued sun exposure. The presence of such lesions, particularly in a patient with a history of extreme photosensitivity, strongly suggests XP and underscores the need for close monitoring for potential skin malignancies. Ocular photosensitivity was quantified using a standardized UV provocation test (Grade 3 erythema after 5 min of simulated sunlight exposure). Symptoms included burning pain (VAS score: 7/10) and conjunctival injection, consistent with XP-associated keratopathy. She noted that her cutaneous lesions began in early childhood, with a history of a similar lesion on her right cheek, surgically excised with flap repair 10 years prior. Notably, her left eye was enucleated at age 12, though the reason could not be recalled.

**Figure 2:**
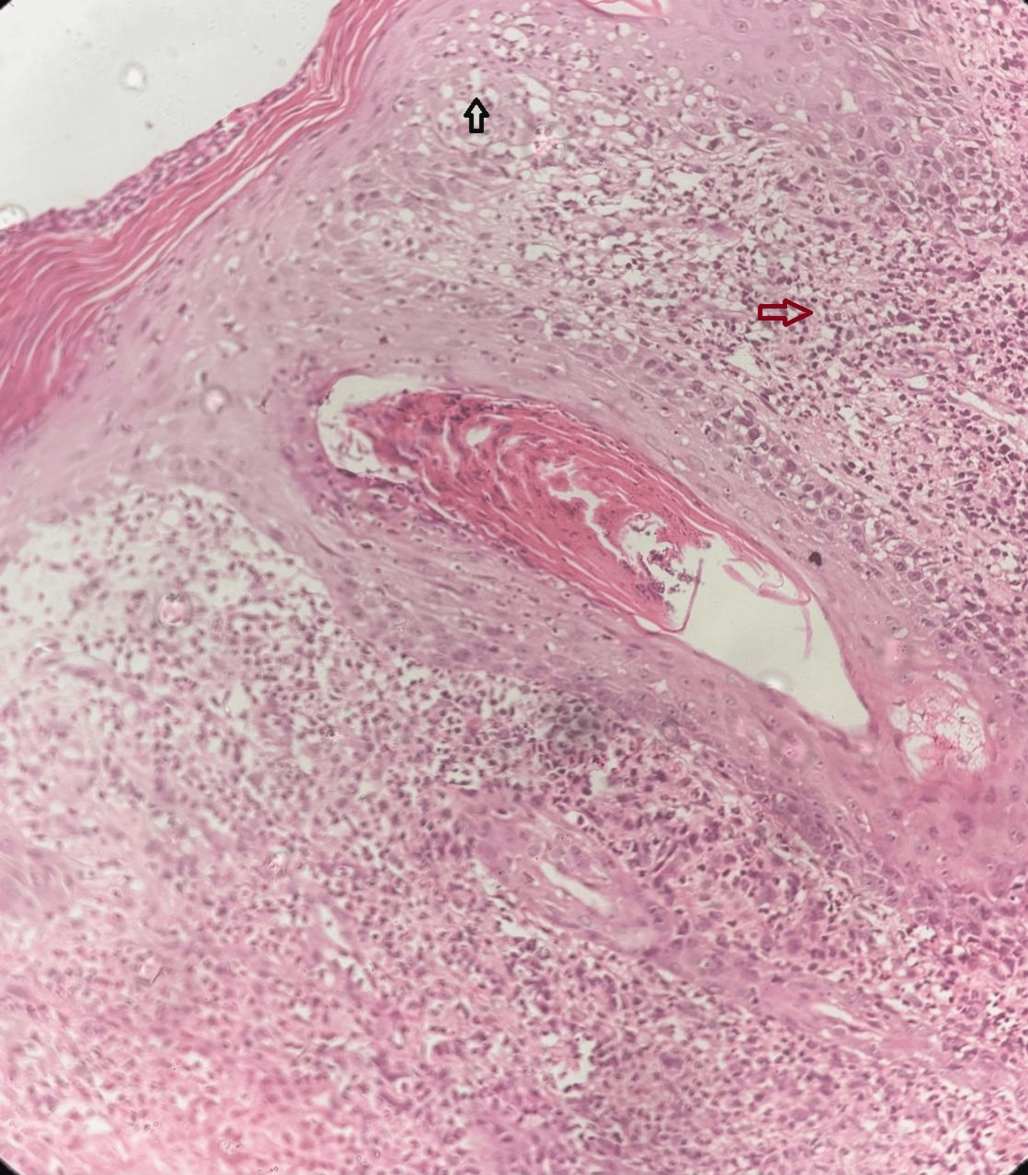
Histopathological examination with haematoxylin and eosin stain at 40x magnification revealed an invasive tumor originating from hyperplastic and dysplastic squamous epithelium, primarily arranged in nests and sheets (black arrow). The tumor cells are significantly anaplastic, showing vesicular nuclear chromatin, prominent nucleoli, and moderate cytoplasm (red arrow).

Among her five siblings, a 38-year-old brother and a 42-year-old sister experienced similar symptoms that began in childhood. However, neither her parents nor her two children exhibited any skin-related issues. There was no history of consanguinity. A biopsy was performed, with differential diagnoses including basal cell carcinoma, squamous cell carcinoma, and malignant melanoma. Histopathological examination revealed an invasive tumor arising from hyperplastic and dysplastic squamous epithelium, predominantly arranged in nests and sheets. The tumor cells were markedly anaplastic, exhibiting vesicular nuclear chromatin, prominent nucleoli, and moderate cytoplasm (Figure 2).

Cytokeratin positivity confirmed the diagnosis of poorly differentiated squamous cell carcinoma. The patient was referred to oncology, neurology, and ophthalmology for further management.

Comparing this case with existing literature on XP-related SCC, it aligns with the well-documented predisposition of XP patients to early-onset skin cancers [3]. The significant increase in risk, particularly in sun-exposed areas, and the early age of diagnosis in this case reflect trends seen in other studies. The patient’s family history also supports the genetic basis of the disease, with several relatives displaying similar symptoms.

XP's impact extends beyond the physical, with significant psychosocial challenges for both the patient and her family. The chronic nature of the disease, coupled with the risk of visible skin changes and the psychological burden of cancer, necessitates a multidisciplinary approach, including psychological support and counseling, to help the patient and her family cope with the emotional toll of the disease. Ongoing care is crucial in improving both the physical and emotional well-being of the patient.

## Discussion

XP presents significant clinical challenges due to its severe photosensitivity and the associated risk of developing multiple skin cancers at a young age. The case of the 55-year-old female patient highlights the urgent need for early diagnosis and proactive management of XP, particularly in populations at risk. The neglect of sun protection and regular medical care can lead to devastating outcomes, including the development of squamous cell carcinoma. Early implementation of sun protection measures could be a crucial factor in enhancing this patient's life expectancy, underscoring the importance of proactive management for those with XP.

Public awareness and education about XP are crucial for improving outcomes. Families affected by XP should be provided with comprehensive information about preventive measures, including the importance of sun avoidance, protective clothing, and regular dermatological check-ups. Establishing specialized clinics for XP patients can facilitate multidisciplinary care, involving dermatologists, oncologists, ophthalmologists, and genetic counselors.

The high incidence of skin cancers associated with XP highlights the necessity for ongoing research into effective treatments and preventive strategies. Genetic counseling can also play a vital role for families with a history of XP, enabling them to understand the implications for future generations [5].

The psychosocial impact of living with XP is significant. Patients often experience emotional challenges due to visible skin changes, frequent medical visits, and the increased risk of skin cancers. Providing psychological support and counseling can help patients and their families cope with the emotional burden, improving treatment adherence and overall quality of life. In this case, the patient reported significant anxiety regarding the recurrence of skin cancers, especially given her history of surgical excisions and the ongoing risk of malignancy. This emotional burden emphasizes the psychosocial challenges faced by individuals with XP and highlights the critical need for psychological support to complement medical care

In summary, though XP is a rare condition with a risk of transmission of 25% if both parents are carriers, its impact on patients and their families is profound. A coordinated approach that emphasizes prevention, early detection, and multidisciplinary care is essential to mitigate the risks associated with this debilitating disorder. Increased awareness within the medical community and society at large can help improve the quality of life for individuals living with XP and reduce the incidence of associated malignancies.

## Conclusions

XP is a rare but serious condition that requires early diagnosis, strict sun protection, and regular medical care to prevent life-threatening complications. This case’s survival to 55 years significantly exceeds the typical life expectancy of 29-37 years for XP patients, suggesting the presence of potential protective factors, such as reduced sun exposure or other unrecognized elements, that may have contributed to this extended survival. Further investigation into these factors could provide valuable insights into improving outcomes for XP patients and help inform strategies for prevention and management.

Given the patient’s late-onset squamous cell carcinoma (SCC) and exceptional survival, targeted prevention strategies (e.g., UV-blocking films at home) and structured psychosocial support, as evidenced by the patient’s reported distress, should complement standard care.

Multidisciplinary management and public awareness are crucial to improving outcomes. Genetic counseling and ongoing research remain essential for supporting affected families and advancing treatment options
